# Bidirectional regulatory effects of exercise on emotional eating in depression: an ERP-based narrative review

**DOI:** 10.3389/fnut.2026.1832858

**Published:** 2026-05-29

**Authors:** Wenshiman Ma, Wenlong Hou, Jingfang Zhai, Haoping Yang, Baofeng Zhang, Li Luo

**Affiliations:** 1 School of Physical Education and Sports Science, Soochow University, Suzhou, China; 2 Prenatal Diagnosis Medical Center of Xuzhou Central Hospital, Xuzhou Clinical College of Xuzhou Medical University, Xuzhou, China; 3 School of Psychology, Beijing Sport University, Beijing, China

**Keywords:** depression, emotional eating (EE), emotional overeating, emotional undereating, event-related potential (ERP), exercise

## Abstract

Depression is frequently accompanied by emotional eating (EE). However, existing research has largely reduced EE to excessive food intake, overlooking the two opposing phenotypes within depressive populations: emotional overeating (EOE) and emotional undereating (EUE). Furthermore, self-report measures are limited in capturing the dynamic neural processes triggered by negative emotions. To address these issues, this narrative review integrated event-related potential (ERP) evidence from studies on depression, EE, and exercise interventions. The neural characteristics of the two EE phenotypes were compared across multiple processing stages, including attentional allocation, conflict monitoring, inhibitory control, and reward processing. The synthesized evidence suggests that EOE may be characterized by exaggerated reward responsiveness coupled with deficient inhibitory control, whereas EUE is marked by attenuated reward processing accompanied by excessive conflict monitoring. To mitigate inferential risks from cross-population and cross-task integration, the reviewed evidence was hierarchically stratified according to inferential distance, and model conclusions were explicitly bounded. Based on this framework, we propose a bidirectional regulatory model in which exercise is hypothesized to modulate neural homeostasis in a baseline-dependent manner. Within this hypothesis-generating framework, exercise may primarily affect putative EOE-related profiles by supporting cognitive control processes and attenuating the motivational salience of food cues, whereas it may affect putative EUE-related profiles by potentially supporting reward responsiveness and attenuating excessive monitoring. These proposed pathways remain to be tested in phenotype-stratified ERP intervention studies. Future research should implement phenotype-stratified randomized controlled exercise trials combined with emotion-induced food-related ERP tasks to test the model predictions and advance personalized intervention strategies.

## 1 Background

Depression is a mental disorder primarily characterized by impairments in emotion regulation. Its clinical manifestations extend beyond persistent low mood and anhedonia to frequently include pronounced disturbances in eating behaviors (1–3). Studies have indicated that approximately 30–50% of individuals with depression report various forms of disordered eating behaviors (4, 5). Among these, emotional eating (EE) represents a prominent behavioral phenotype in depressive populations; it refers to eating in response to negative emotions or stress rather than to physiological hunger as a means of emotion regulation (6). Previous studies have consistently demonstrated a significant positive association between the severity of depressive symptoms and the prevalence of EE (7, 8). This association has been linked to deficits in emotion regulation capacity and impaired interoceptive processing (9). Although EE may provide transient affective relief, it often exacerbates depressive symptoms through maladaptive cycles of post-eating shame, guilt, and body anxiety, thereby increasing disease burden and recurrence risk (3, 10).

However, there has been a tendency in existing research to simplify EE as unidirectional excessive eating, thereby neglecting the substantial heterogeneity in eating behaviors among individuals with depression (11). Accumulating evidence has indicated that the impact of depression on EE is bidirectional. A subset of individuals exhibit emotional overeating (EOE), which is characterized by reward-driven approach behaviors, whereby eating as a result of a negative emotional state serves as a source of hedonic relief (2, 3, 12). By contrast, another subgroup demonstrates emotional undereating (EUE), characterized by emotion-triggered reductions in eating behavior, diminished desire to eat, or eating avoidance, which may be informed by blunted reward responsiveness or anhedonia-related mechanisms (2, 3, 13–15). Importantly, “changes in appetite or weight” within the diagnostic criteria for depression primarily reflect alterations driven by physiological or metabolic processes, while EE emphasizes dysregulation triggered by negative emotions; thus, these two constructs are not conceptually or mechanistically equivalent. In this review, EUE is operationally defined as a pattern in which negative affect, stress, or emotion-regulation difficulty is followed by reduced food intake, diminished desire to eat, or avoidance of eating. This phenotype differs from general depressive appetite loss, which may occur without an identifiable emotional trigger or regulatory function and may reflect broader vegetative, metabolic, or somatic symptoms of depression. Similarly, anhedonia is treated as an adjacent but non-equivalent construct: it may help explain reduced reward responsiveness in EUE, but it is not itself sufficient to define EUE unless it is linked to emotion-triggered reductions in eating behavior. Accordingly, appetite loss and anhedonia were used in this review as mechanistic and inferential sources only when they informed the interpretation of EUE-related reward attenuation or eating avoidance, rather than as direct evidence of EUE. This bidirectional divergence in behavioral phenotypes suggests the existence of distinct neurocognitive processing pathways underlying eating behaviors in individuals with depression.

To reveal these differentiated neural mechanisms, reliance solely on traditional structured self-report scales—such as the Three-Factor Eating Questionnaire (TFEQ) (16), the Dutch Eating Behavior Questionnaire (17), and the Adult Eating Behavior Questionnaire (18) are often insufficient to capture the dynamic cognitive processes underlying eating behaviors. By contrast, event-related potentials (ERPs), with their millisecond-level temporal resolution, provide a unique perspective for dissecting the processing of food cues in depressed individuals (19, 20). Based on existing evidence, distinct eating phenotypes may exhibit partly divergent neural time courses. In the early attentional stage, EE behaviors are often accompanied by enhanced attentional bias, as evidenced by increased early positive waves (21, 22). In the conflict monitoring stage, available indirect evidence from food cue and inhibitory control studies has suggested that putative EOE-related profiles may involve reduced N2 and P3 amplitudes, potentially reflecting weakened inhibitory control recruitment (23, 24), whereas putative EUE-related profiles may exhibit enhanced or dysregulated amplitudes, potentially suggesting excessive conflict monitoring (25). Subsequent differences are further reflected in the late reward evaluation stage: EOE tends to show enhanced LPP, reflecting heightened motivational salience or reward sensitivity, whereas anhedonia-related reward blunting, which may partially inform the EUE phenotype, may be reflected in attenuated LPP responses (22, 26, 27). However, the majority of existing ERP studies have focused on healthy or overweight groups, while systematic electrophysiological evidence targeting this heterogeneity of EE within depressive populations remains scarce.

Addressing these complex neurocognitive characteristics, current pharmacological treatments and cognitive behavioral therapy (CBT) have certain limitations, such as side effects and limited efficacy in regulating automated eating responses (28). Against this backdrop, exercise has emerged as a safe, low-cost, non-pharmacological intervention and has been confirmed to effectively improve depressive symptoms and related cognitive control functions (29, 30). Previous electrophysiological studies have further suggested that exercise interventions may modulate N2 and P3 amplitudes related to inhibitory control and may also influence LPP amplitudes involved in emotional arousal and motivational processing (31–36). Based on this evidence, this review proposes a bidirectional regulatory hypothesis of exercise: specifically, exercise may exert regulatory effects on EE via distinct neural pathways. For individuals hypothesized to exhibit EOE, exercise may support inhibitory control processes, potentially reflected by changes in N2 and P3, and may attenuate sensitivity to highly rewarding food cues as a hypothesis-generating possibility (35, 37). In contrast, for individuals hypothesized to exhibit EUE, exercise may support reward-related responsiveness, potentially reflected in changes in LPP, thereby providing a possible pathway for alleviating anhedonia-related reward blunting rather than directly treating anhedonia itself (3, 38).

In summary, the majority of existing studies have examined the relationships between exercise, depression, and eating behavior in isolation, lacking an integrated perspective that considers the heterogeneity of emotional eating. Therefore, this review aims to distinguish EE into two phenotypes, EOE and EUE, review and integrate existing ERP electrophysiological evidence, and explore in depth the bidirectional regulatory mechanisms of exercise intervention on eating behaviors in depressed individuals. On this basis, this article attempts to construct a “Bidirectional Neurocognitive Model of Exercise Regulation,” exploring how exercise may modulate different forms of eating abnormalities through distinct neural pathways. This will not only help clarify the refined neural pathways through which exercise improves depressive symptoms but also provide a theoretical basis for developing phenotype-targeted intervention strategies in the future.

## 2 Methods

### 2.1 Data sources and search strategy

This study adopted a narrative review approach to identify literature related to depression, emotional eating phenotypes, food-cue processing, ERP-based neurocognitive indices, and exercise-related neural regulatory mechanisms. The search covered the period from database inception to 17 February 2026. The databases searched were PubMed, Web of Science, Embase, and the Cochrane Library. To comprehensively capture the interdisciplinary focus of this review, the search strategy was organized around five complementary thematic blocks: depression and emotional eating phenotypes; ERP studies of food cues and emotional eating; ERP studies of depression-related reward processing and cognitive control; ERP studies of exercise in relation to depression or eating behavior; and a supplementary comprehensive search. The full search strings and the initial number of records retrieved for each search block are provided in the Supplementary material. The first four blocks were not intended to be mutually exclusive or additive evidence categories; rather, they were designed to retrieve literature relevant to different components of the conceptual model. The fifth block served as a comprehensive, restrictive search for direct evidence, integrating all core concepts.

The initial database search identified 27,031 records from PubMed, Web of Science, Embase, and the Cochrane Library. After duplicate records were removed through DOI, PMID, database accession number, and normalized-title matching, 12,905 unique records remained for title and abstract screening.

### 2.2 Inclusion and exclusion criteria

The inclusion criteria were as follows:

1. The study topic was related to depression, depressive symptoms, or depression-related emotion regulation abnormalities, including mechanisms such as anhedonia, altered reward sensitivity, emotion regulation deficits, cognitive control dysfunction, conflict monitoring, or error monitoring.
2. The study addressed emotional eating or related eating phenotypes, including emotional overeating, emotional undereating, overeating, undereating, appetite changes, food cravings, food reward, or food-cue processing.
3. The study provided evidence for the neurocognitive processes underlying emotional eating, with priority given to studies reporting ERP indices. ERP studies served as the core evidence for model construction in this review. Particular attention was given to components such as P1/N1, N2, P3, LPP, SPN, RewP, ERN, and Pe, along with the corresponding processes of attentional allocation, conflict monitoring, inhibitory control, reward anticipation, reward evaluation, reward feedback, and error monitoring.
4. The study examined exercise, physical activity, and exercise-related neural regulatory mechanisms to inform the potential effects of exercise on depression, eating behavior, cognitive control, reward processing, conflict monitoring, error monitoring, and emotion regulation.
5. Given the limited number of food-related ERP studies directly examining emotional overeating and emotional undereating in populations with depression, some non-ERP studies were also included, but only when they supported the definition of emotional eating phenotypes, interpretation of ERP components, or construction of exercise-related regulatory mechanisms. These studies included behavioral studies, neuroimaging studies, systematic reviews, theoretical models, methodological papers, and measurement studies. These sources were not treated as direct human ERP evidence but rather as auxiliary or explanatory evidence.
6. Animal studies and basic neuroscience studies were included only when they helped explain exercise, food motivation, reward systems, dopamine, BDNF, striatal function, or depression-related neural mechanisms. These studies were used only as inferential evidence and were not treated as direct evidence for the proposed human ERP model.
7. The study type was an Article or a Review, and the language was English.

The exclusion criteria were as follows:

1. Non-English publications, conference abstracts, editorials, letters, book reviews, study protocols, non-peer-reviewed literature, or records with insufficient information for screening.
2. Studies with no clear relevance to depression, depressive symptoms, or depression-related abnormalities in emotion regulation.
3. Studies with no clear relevance to emotional eating, eating behavior, appetite changes, food cravings, food reward, or food cue processing.
4. Studies that did not report ERP indices and could not support the interpretation of ERP components, the definition of emotional eating phenotypes, or the construction of exercise-related regulatory mechanisms.
5. Studies with no clear relevance to exercise, physical activity, or exercise-related neural regulatory mechanisms.
6. Studies that focused only on general nutritional intake, dietary composition, weight management, metabolic outcomes, exercise performance, or general cognitive function and could not serve the framework of “depression–emotional eating–ERP–exercise regulation” in this review.
7. Animal studies or basic neuroscience studies were excluded if they could not explain exercise, food motivation, reward systems, dopamine, BDNF, striatal function, or depression-related neural mechanisms. Even when mechanistically relevant, these studies were used only as inferential evidence and were not assigned to the direct human ERP evidence level.
8. Duplicate publications or duplicate records.

### 2.3 Literature screening process

Based on the inclusion and exclusion criteria described above, two reviewers independently conducted the initial screening of titles, abstracts, and keywords. Records with insufficient information in the title or abstract or with unclear relevance in terms of study population, mechanisms, or outcomes, were retained for manual rechecking rather than being excluded at this stage. Disagreements regarding eligibility were resolved through discussion. When consensus could not be reached, a third reviewer was consulted to make the final decision. After this initial screening, 141 records were marked as preliminarily relevant to the topic, 789 records were marked for further manual rechecking because of insufficient information in the title or abstract or unclear relevance of the study population, mechanisms, or outcomes, and 11,975 records were excluded because they did not show sufficient relevance to the “depression–emotional eating–ERP–exercise-related modulation” framework at the level of the title, abstract, or keywords.

Subsequently, the 930 records that were either preliminarily retained or required manual rechecking were further screened and classified through full-text reading, evidence-tier criteria, citation chasing, and manual supplementation. Full-text eligibility assessment and evidence-tier assignment were also independently checked by two reviewers. Any disagreement regarding inclusion, exclusion, or evidence-tier classification was resolved through discussion, and unresolved disagreements were adjudicated by a third reviewer. During the full-text assessment, the reviewers focused on whether each study could support one or more of the following aspects: the definition of depression-related emotional eating phenotypes; ERP evidence for food-cue or food-reward processing; depression-related reward processing, cognitive control, conflict monitoring, or error monitoring mechanisms; the regulatory effects of exercise on emotion, eating behavior, or neurocognitive processes; and the relevance of this evidence to the construction of the proposed model. After manual rechecking, full-text eligibility assessment, evidence-tier assignment, and necessary citation chasing, 874 records were excluded, and 56 studies were finally identified as the core evidence base for evidence-tier synthesis and model construction.

**Figure 1:**
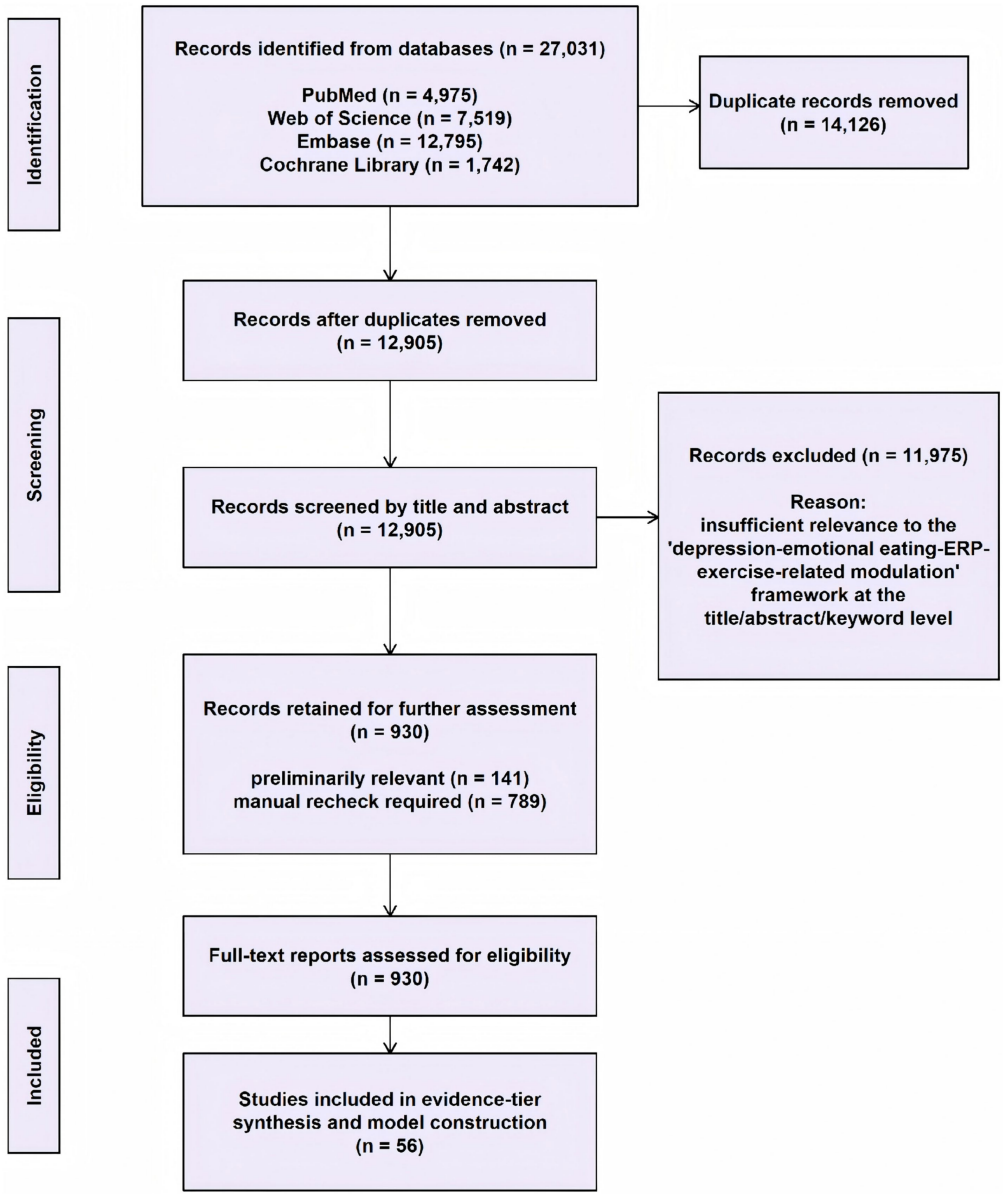
Flowchart of literature search and study selection.

It should be noted that the total number of references cited in this review exceeds 56. The 56 studies refer to the core evidence base included in the evidence-tier synthesis and model construction rather than to the total number of references cited throughout the manuscript. In addition to these core evidence studies, this review also cites background, theoretical, methodological, scale-related, and mechanistic references to support the introduction, conceptual definitions, ERP component interpretation, exercise-related regulatory mechanisms, and discussion sections (see Figure 1).

### 2.4 Evidence-tier classification

To avoid treating evidence from different sources, populations, and task paradigms as equivalent, the final studies included in the comprehensive synthesis were classified into three tiers according to their proximity to the research question: direct evidence, indirect evidence, and inferential evidence. Evidence tiers were assigned based on four criteria: whether the study population involved individuals with depression or depressive symptoms; whether the study addressed emotional eating or EOE/EUE phenotypes; whether the task involved food cues, emotion induction, reward processing, inhibitory control, or error monitoring; and whether the study reported ERP components or could explain the neurocognitive processes corresponding to ERP components. Direct evidence served as the core basis for model construction; indirect evidence was used to supplement mechanistic explanations across populations or task paradigms; and inferential evidence was used only to support theoretical pathways and biological plausibility (see Table 1).

**Table 1:** Evidence-tier classification criteria.

Evidence tier	Classification criteria	Main use	Interpretive boundary
Direct evidence	Depressive or depressive-symptom samples; reported ERP indices; tasks involving food cues, emotion induction, reward processing, inhibitory control, conflict monitoring, or error monitoring; studies distinguishing EOE/EUE phenotypes were prioritized	To support the ERP temporal framework, core component interpretation, and main model pathways	Can serve as core evidence for the model, but task and sample differences still need to be considered
Indirect evidence	Non-depressive samples, or studies without EOE/EUE stratification, but involving food cues, emotional eating, obesity, binge-eating disorder, restrained eating, exercise intervention, reward processing, or cognitive control; may include ERP, EEG, fMRI, or behavioral studies	To supplement direct evidence and support local mechanistic interpretation	Cannot be directly equated with ERP characteristics of EOE/EUE in populations with depression
Inferential evidence	Theoretical models, reviews, methodological papers, measurement studies, animal studies, or basic neuroscience studies that could explain ERP components, exercise-related regulatory mechanisms, or biological plausibility	To support conceptual definition, theoretical pathways, and mechanistic plausibility	Not treated as direct human ERP evidence; animal and basic neuroscience studies were only used as mechanistic inferential support

Direct evidence was used to support the core ERP components and neurocognitive stages in the model. Studies without EOE/EUE stratification were not treated as direct phenotype-specific evidence. Indirect evidence was used to support cross-population or cross-task mechanistic inference. Inferential evidence was only used to explain conceptual origins, theoretical pathways, and biological plausibility. Conclusions based on indirect or inferential evidence were described cautiously and explicitly labeled in the evidence tables to avoid overinterpreting non-ERP studies, non-depressive samples, or animal findings as direct ERP evidence for depression-related emotional eating.

### 2.5 Quality appraisal and risk-of-bias considerations

Because this review was designed as a narrative and theory-building synthesis rather than a systematic review or meta-analysis, a formal risk-of-bias assessment using standardized tools was not conducted. Instead, the methodological relevance and inferential strength of each study were considered during full-text assessment and evidence-tier assignment. Particular attention was given to sample characteristics, phenotype specificity, task relevance, ERP component specificity, and whether the evidence was derived from depressive populations, non-depressive clinical or community samples, reviews, animal studies, or theoretical models.

Studies with greater inferential distance from depression-related EOE or EUE were not used as direct evidence, and their contribution was restricted to mechanistic interpretation or biological plausibility. This approach was intended to reduce overinterpretation across populations, tasks, and evidence types. However, the absence of a formal risk-of-bias assessment remains a limitation of the present narrative review, and this should be considered when interpreting the proposed model.

Importantly, the evidence-tier classification used in this review was not intended to function as a methodological quality rating or as a substitute for formal risk-of-bias assessment. Rather, it was designed to describe the inferential distance between each source of evidence and the central question of this review. Thus, studies classified as indirect or inferential evidence were not necessarily of lower methodological quality; instead, they provided evidence from different populations, task contexts, stimulus categories, or levels of analysis. Conversely, studies classified as direct evidence were considered more proximal to the proposed model but were still interpreted in light of sample characteristics, task design, ERP component specificity, and other methodological constraints. Therefore, the evidence-tier system should be understood as an inferential-distance framework rather than a quality appraisal framework.

### 2.6 ERP component interpretation framework

Because ERP components are not process-pure markers, their interpretation in this review was constrained by paradigm type, sample characteristics, stimulus category, temporal window, and analytic contrast. The same ERP component may reflect different neurocognitive processes across food-cue viewing tasks, inhibitory-control paradigms, reward-anticipation tasks, reward-feedback paradigms, and error-monitoring tasks. Therefore, before conducting the phenotype-level synthesis, we summarized the paradigm- and context-specific interpretation of each ERP component included in the proposed model. This step was intended to avoid treating components such as N2, P3, LPP, SPN, RewP, ERN, and Pe as invariable markers of a single cognitive function across different task contexts. Table 2 presents the main task context, sample source, food versus non-food context, summarized component direction, and inferential limit for each ERP component.

**Table 2:** Paradigm- and context-specific interpretation of ERP components included in the model.

ERP	Main task context	Main evidence source	Stimulus context	Direction summarized in this review	Inferential limit
P1/N1	Passive viewing; attentional bias; early food-cue processing	BED, obesity, high EE, or non-depressive samples	Mainly food cues	Increased P1/N1: early food-cue capture in EOE	Indirect evidence; not depressive EOE-specific
N2	Inhibitory control; conflict monitoring; Go/No-Go	EE, obesity, depression, or non-stratified samples	Food and non-food	Reduced N2: insufficient control in EOE; increased/dysregulated N2: excessive monitoring in EUE	Not process-pure; direction is task-dependent
P3	Go/No-Go; inhibition; reward evaluation; exercise-cognition tasks	Food cue, depression, exercise, or non-stratified samples	Food and non-food	Reduced P3: insufficient control/resource allocation; increased P3 after exercise: possible enhanced resource recruitment	Context-dependent index of cognitive resource allocation
LPP	Food cue viewing; emotional viewing; motivation; emotion regulation	Food cue, high EE, obesity, depression, or exercise studies	Mainly food/emotional stimuli	Enhanced LPP: motivational salience in EOE; attenuated LPP: reward blunting in EUE	Depends on stimulus valence, task goal, and time window
SPN	Reward anticipation	Depression, anhedonia, or reward-processing studies	Primarily non-food rewards	Reduced SPN: insufficient reward anticipation in EUE	Inferential for EUE; limited food-specific evidence
RewP	Reward feedback	Depression, anhedonia, or reward-feedback studies	Primarily non-food rewards	Attenuated RewP: blunted reward feedback in EUE	Inferential when transferred to food-related EUE
ERN/Pe	Error monitoring; performance monitoring	Depression and error-monitoring studies	Primarily non-food control tasks	Increased ERN/reduced Pe: high monitoring with poor post-error adjustment in EUE	Inferential for eating avoidance; not food-specific

BED, binge eating disorder; EE, emotional eating; EOE, emotional overeating; EUE, emotional undereating; ERN, error-related negativity; LPP, late positive potential; Pe, error positivity; RewP, reward positivity; SPN, stimulus-preceding negativity. ERP interpretations were constrained by task, sample, stimulus type, time window, and analytic contrast. Non-depressive, non-food, non-ERP, animal, review, or non-EOE/EUE-stratified evidence was classified as indirect or inferential rather than direct phenotype-specific evidence.

## 3 Neural electrophysiological correlates of EE phenotypes

### 3.1 Neural electrophysiological characteristics of EOE

There are still relatively few ERP studies that have directly investigated individuals with depression and EOE using emotion-induced eating paradigms or food cue-based tasks. Consequently, the following characterization of EOE-related electrophysiological features is largely derived from ERP evidence in populations with BED, obesity, or elevated EE levels, with conditional extrapolation to depressive EOE phenotypes at the mechanistic level. EOE is highly prevalent among individuals with depression and is conceptualized as a functional imbalance arising from the interaction of heightened reward system responsivity, impaired inhibitory control, and disrupted emotion regulation processes (7, 39–41). In the context of food cue processing, indirect evidence from studies involving elevated emotional eating traits, BED, and obesity-related eating dysregulation suggests heightened sensitivity to highly palatable, high-calorie foods. A substantial body of indirect evidence from these studies has reported enhanced neural responses to high-calorie food cues compared with healthy controls (42–44). Specifically, findings from studies involving elevated emotional eating traits and obesity-related eating dysregulation have suggested enhanced early attentional allocation to food cues, as indexed by increased amplitudes of early ERP components such as P1 and N1 (45), which may reflect heightened motivational salience during early-stage perceptual processing (46). At the level of conflict monitoring, indirect evidence from emotional eating and related food control studies has reported reduced N2 amplitudes (47), which may reflect insufficient recruitment of conflict-monitoring resources. Consistent with this pattern, ERP studies involving elevated emotional eating traits, BED, or obesity-related eating dysregulation have reported increased P3 and LPP amplitudes in response to high-calorie food images, which may reflect stronger attentional engagement and motivational processing elicited by food cues (42, 48). Additionally, deficits in prefrontal cortex (PFC)-mediated inhibitory control have been consistently observed in individuals exhibiting EOE traits. The proposed “monitoring–execution” dual-loop dysfunction model of PFC control processes (49) may contribute to difficulties in initiating behavioral inhibition when individuals are confronted with highly salient food cues. In Go/No-Go paradigms, a canonical measure of response inhibition, food-related inhibitory-control studies involving elevated emotional eating traits, BED, and obesity-related eating dysregulation have reported reduced No-Go P3 amplitudes during successful inhibition relative to healthy controls (43), which may reflect diminished recruitment of inhibitory control processes. These attenuated inhibitory-related ERP responses may indicate reduced engagement of inhibitory control when individuals are confronted with highly salient food cues. During later stages of motivational and emotional processing, enhanced LPP responses to food cues have been reported in studies involving emotional eating, obesity-related eating dysregulation, and depression-related alterations in food-cue processing (22, 42). This pattern may reflect prolonged motivational engagement with emotionally salient food stimuli. In addition to altered reward and inhibitory processing, EOE-or binge-eating-related mechanisms have also been associated with emotion-regulation difficulties (50). Under negative affect, heightened emotional reactivity combined with insufficient regulatory control increases reliance on eating as an immediate coping strategy, thereby reinforcing a maladaptive cycle of negative emotions, eating to experience relief, and behavioral reinforcement (51–53). Taken together, the indirect ERP evidence available from food cues, emotional eating, BED, obesity, and non-depressive samples suggests that EOE in depressive contexts may involve a functional imbalance characterized by heightened food-related motivational salience, insufficient cognitive control recruitment, and difficulty regulating emotions. However, this profile should be interpreted as a mechanistic extrapolation rather than as direct ERP evidence from depressive EOE populations. A stratified overview of ERP evidence related to EOE is provided in Table 3.

**Table 3:** Stratified overview of ERP evidence related to EOE.

Processing stage	ERP	Putative ERP features	Primary neurocognitive interpretation	Evidence level	References
Early attention	P1/N1	Increased food-cue attentional bias	Increased early attentional capture and food-cue motivational salience	Indirect evidence	(21, 22, 44, 48)
Conflict monitoring	N2	Reduced N2 during inhibition	Insufficient recruitment of neural resources for conflict detection	Indirect evidence	(23, 47)
Behavioral inhibition	P3	Attenuated P3 during inhibition	Insufficient PFC execution control	Indirect evidence	(24, 43)
Late motivation/emotion	LPP	Enhanced food-cue LPP	Possible heightened reward motivation and sustained attention	Indirect and inferential evidence	(22, 26, 42, 48)
Emotion regulation-related mechanisms	—	Amplified negative affect and food reward responses	Eating as an emotional regulation strategy	Inferential evidence	(51, 52)

BED, binge eating disorder; EE, emotional eating; EOE, emotional overeating; LPP, late positive potential; ERP, event-related potential; PFC, prefrontal cortex. No studies included in this table meet the criteria for direct evidence of ERP characteristics in depressive EOE populations using emotion-induced food-related paradigms. Evidence was therefore classified as indirect or inferential according to population relevance, phenotype specificity, task relevance, and ERP component specificity. Rows without a specific ERP component reflect non-ERP mechanistic inference rather than electrophysiological evidence.

### 3.2 ERP characteristics of EUE in depressive populations

Compared with EOE, studies that have directly investigated individuals with depression with EUE traits using emotion-induced eating paradigms or food cue tasks to systematically characterize ERP features are exceedingly scarce. Accordingly, the present section on the neural electrophysiological mechanisms of EUE is primarily based on ERP evidence related to depression-associated anhedonia, reduced appetite, and reward-processing deficits, with inferential integration used to generate hypotheses about the EUE phenotype. It is important to distinguish these adjacent constructs. General depressive appetite loss refers to reduced appetite or weight change that may occur as part of vegetative or somatic symptoms, and it does not necessarily involve an identifiable emotional trigger (54, 55). Anhedonia refers to diminished pleasure or reward responsiveness and may contribute to reduced motivation to eat, but it does not by itself constitute EUE (13, 56, 57). In contrast, EUE specifically refers to emotion-triggered reductions in eating behavior, diminished desire to eat, or avoidance of eating. Therefore, evidence from anhedonia, reduced appetite, and reward-processing studies was used here only as indirect or inferential support for EUE-related mechanisms, rather than as direct phenotype-specific ERP evidence. Notably, EUE should not be considered a simple reduction in appetite. In the present review, EUE is conceptualized as an emotion-triggered reduction in eating behavior that may be partly informed by indirect evidence of attenuated reward processing and heightened conflict monitoring. With respect to motivational and reward-related processing, the available indirect and inferential evidence suggests that EUE may involve attenuated reward-related mobilization rather than a simple reduction in appetite. This putative pattern may be reflected by reduced neural engagement in value evaluation, attentional allocation, and reward feedback when food cues or reward-related stimuli are processed. During the reward anticipation stage, reduced stimulus-preceding negativity (SPN) has been reported in depression-related reward processing studies, suggesting difficulty in mobilizing anticipatory resources before reward delivery (58, 59). During reward evaluation and attentional allocation, reduced P3 amplitude may indicate insufficient cognitive resource allocation, whereas sustained attenuation of LPP may suggest reduced motivational salience of appetitive or food-related cues (60, 61). However, because these findings are drawn largely from studies on depression, anhedonia, reduced appetite, or general reward processing rather than from EUE-stratified depressive samples, this pattern should be interpreted as a hypothesis-generating account of EUE-related reward attenuation rather than as direct, phenotype-specific ERP evidence.

At the reward feedback stage, EUE may be associated with difficulty in generating robust positive neural responses to food-related outcomes, potentially reflected by attenuated reward positivity (RewP). However, this inference is derived primarily from depression- and anhedonia-related reward-feedback studies rather than from EUE-stratified food-related ERP research. Although direct investigations of eating-related RewP in EUE remain limited, RewP has been widely implicated as a neural marker of anhedonia and impaired reward prediction error signaling, with primary contributions from striatal pathways (54, 62–66). Accordingly, blunted RewP provides important indirect evidence for a diminished reward experience in EUE.

Beyond attenuated reward processing, the neural profile of EUE cannot be understood as a purely passive consequence of motivational blunting. In addition to an overall reduction in motivational drive, EUE may involve a more active inhibitory or avoidance-oriented tendency, although this interpretation remains inferential and requires direct validation in depressive samples stratified by EUE, which is characterized by abnormally heightened activation within conflict monitoring-related neural circuits. This pattern may arise from internal conflict between physiological eating demands and emotion-driven avoidance. During conflict processing, EUE-related eating avoidance may be accompanied by enhanced N2 amplitudes, increased error-related negativity (ERN), and reduced error positivity (Pe) (25, 67). However, this pattern is mainly inferred from depression-related conflict- and error-monitoring evidence rather than from direct EUE-specific food-related ERP studies. Enhanced ERN may indicate heightened ACC-related sensitivity to errors or conflict (68), whereas reduced Pe may reflect inefficient post-error adjustment. Together, these findings suggest a possible profile of high monitoring but low regulatory updating, in which eating-related decisions may be more readily experienced as conflictual or avoidant under negative emotional conditions. This interpretation remains hypothesis-generating and requires direct validation in samples of individuals with depression stratified by EUE.

In the context of depression, EUE can be conceptualized as a putative network-level disturbance involving attenuated reward motivation and heightened conflict monitoring. Available indirect evidence from studies on depression-related anhedonia, reduced appetite, reward processing, and error monitoring has suggested possible attenuation across the reward anticipation, evaluation, and feedback stages, including reduced SPN, P3, LPP, and RewP responses (58–66, 69–71). In parallel, enhanced N2 or ERN responses, together with reduced Pe, may reflect heightened conflict or error monitoring with relatively inefficient post-monitoring adjustment (25, 67, 68, 72). However, because these findings have not been systematically demonstrated in depressive samples stratified by EUE, this electrophysiological profile should be regarded as a hypothesis-generating account rather than as a direct phenotype-specific ERP pattern. A stratified overview of the ERP evidence related to EUE is presented in Table 4.

**Table 4:** Stratified overview of ERP evidence related to EUE.

Processing stage	ERP	Putative ERP features	Primary neurocognitive interpretation	Evidence level	References
Reward anticipation	SPN	Reduced SPN amplitude	Insufficient initiation of reward anticipation	Indirect/inferential evidence	(58, 59)
Reward evaluation and attentional allocation	P3	Reduced P3 amplitude	Insufficient mobilization of cognitive resources	Indirect evidence	(60, 61)
Late motivational and emotional processing	LPP	Sustained attenuation of LPP	Insufficient attribution of reward salience	Indirect ERP evidence	(60, 61)
Reward feedback	RewP	Markedly attenuated RewP	Blunted reward feedback processing	Indirect/inferential evidence	(62–66)
Conflict monitoring	N2	Enhanced N2 amplitude	Excessive conflict monitoring	Indirect/inferential evidence	(25)
Error monitoring	ERN/Pe	Increased ERN combined with reduced Pe	Hypervigilant monitoring with impaired post-error adjustment	Indirect/inferential evidence	(67, 68, 72)
Emotion regulation- related mechanisms	—	Active suppression of eating behavior	Eating avoidance driven by emotion-related conflict	Inferential evidence	(13, 56, 57)

SPN, stimulus-preceding negativity; ERN, error-related negativity; EUE, emotional undereating; LPP, late positive potential; ERP, event-related potential; RewP, reward positivity; Pe, error positivity. No studies included in this table meet the criteria for direct evidence of ERP characteristics in depressive EUE populations using emotion-induced food-related paradigms. Evidence was therefore classified as indirect or inferential according to population relevance, phenotype specificity, task relevance, and ERP component specificity. Evidence derived from depression-related anhedonia, reduced appetite, or reward-processing studies was treated as inferential when it was used to characterize EUE-specific mechanisms.

### 3.3 Comparative ERP profiles of the two EE phenotypes in depressive populations

**Figure 2:**
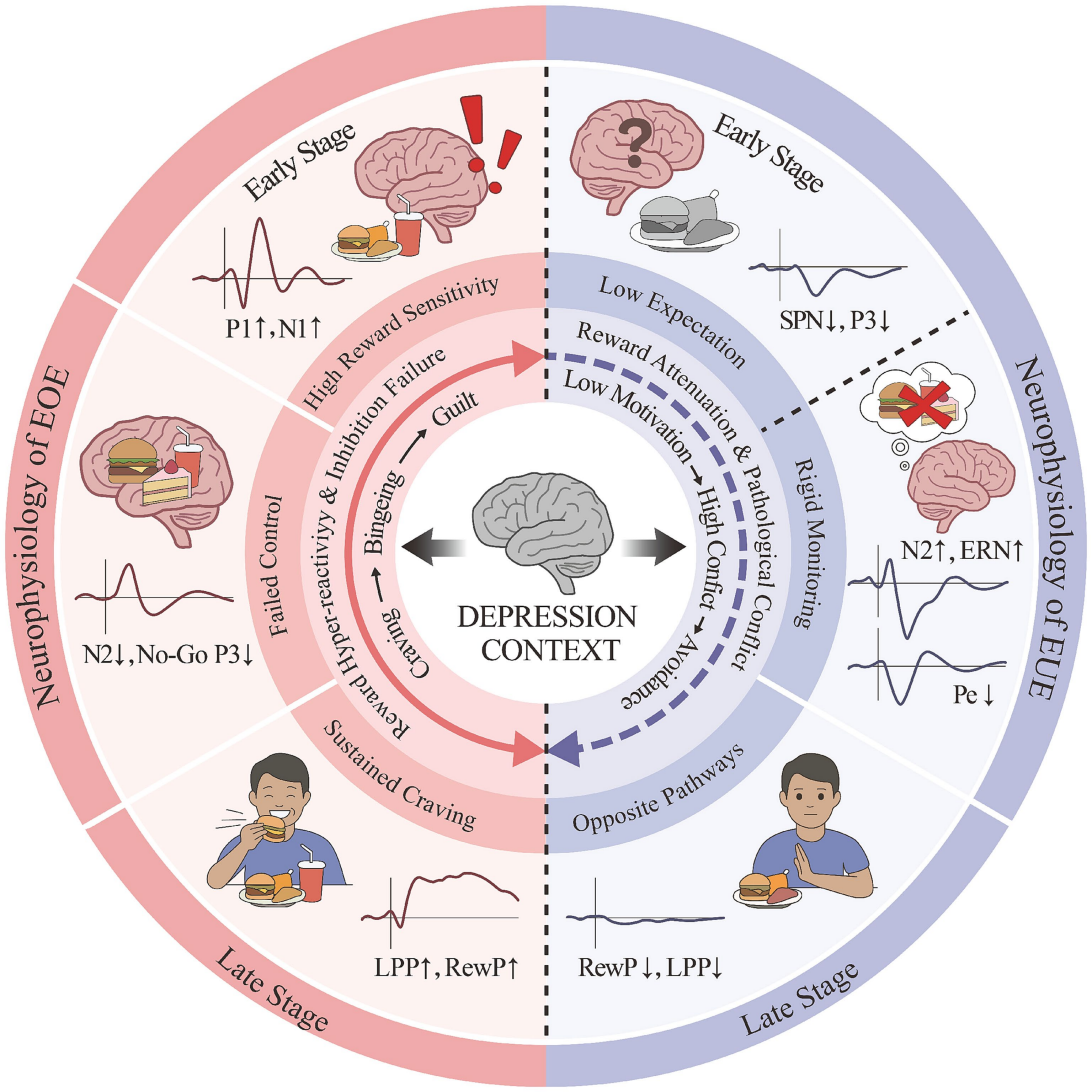
Putative comparative neurophysiological profiles of EOE and EUE in depression. This figure represents a hypothesis-generating synthesis rather than a directly validated ERP model.

By synthesizing the available direct, indirect, and inferential evidence, we propose that EOE and EUE may exhibit directionally divergent electrophysiological profiles within a shared neural processing framework (Figure 2) (6, 13, 39–41, 54–57). These putative profiles are intended to summarize possible forms of functional imbalance in emotion–eating regulation rather than represent directly validated phenotype-specific ERP signatures.

From a global processing perspective, available ERP studies on food cues and emotional eating have suggested that EOE may be associated with amplified food-related motivational processing and relatively insufficient control resource recruitment. This putative profile is most consistently supported for early attentional engagement and late motivational processing, reflected by increased P1/N1 or LPP responses in food cue paradigms, whereas evidence for other reward feedback or error monitoring components remains limited. Beyond the evaluative stage, reward feedback processing may also be relevant to EOE, although direct evidence remains limited. Based on broader reward processing evidence, heightened sensitivity to palatable food rewards may theoretically reinforce negative reinforcement cycles, whereby transient pleasure from eating temporarily alleviates negative affect and strengthens the association between emotional distress and overeating behaviors (52, 64). In parallel, attenuated N2 responses during conflict monitoring and insufficient P3 recruitment during behavioral inhibition indicate an excessive reward drive coupled with constrained inhibitory regulation (22, 26, 42, 47). Under conditions of negative affect, these reward-related effects are further potentiated, causing eating to be more readily adopted as a strategy for emotional relief (51, 52).

In contrast, EUE may involve attenuated reward-related mobilization in combination with heightened conflict or error monitoring. This interpretation is based primarily on depression-related anhedonia, reduced appetite, reward processing, and error monitoring evidence rather than on direct EUE-stratified food-related ERP studies. Within this inferential framework, reduced SPN, P3, LPP, and RewP responses may indicate diminished reward anticipation, reward evaluation, motivational salience, and feedback processing, whereas enhanced N2 or ERN responses with reduced Pe may indicate heightened monitoring with inefficient post-error adjustment (58–68, 72). As a result, eating may be more likely to be processed as conflictual or avoidant in EUE, although this interpretation remains hypothesis-generating and requires direct validation in depressive samples stratified by EUE. Accordingly, the two EE phenotypes should not be interpreted as simple differences in degree along a single continuum. Instead, available evidence suggests that they may reflect divergent functional imbalance pathways, with reward amplification and relatively insufficient control in EOE and reward attenuation with heightened monitoring in EUE. This contrast remains hypothesis-generating and highlights the need for direct ERP studies in depressive samples stratified by EOE and EUE. Because the available ERP literature is not evenly distributed across the two phenotypes, the following comparison should not be interpreted as a symmetrical component-by-component model. Components such as P1/N1, N2, P3, and LPP have relatively stronger relevance for EOE-related food cue and inhibitory control processes, whereas SPN, RewP, ERN, and Pe are used primarily to characterize EUE-related reward attenuation and monitoring abnormalities on the basis of depression- and anhedonia-related evidence. Therefore, components were retained in the comparative table only when they clarified phenotype-specific hypotheses, and cells with insufficient evidence were explicitly marked as such. A comparative overview of the putative ERP profiles associated with EOE and EUE is presented in Table 5.

**Table 5:** Comparative synthesis of putative EOE and EUE neurocognitive profiles.

Comparative dimension	Putative EOE profile	Putative EUE profile	Comparative implication
Dominant emotion–eating pattern	Negative affect may increase food approach and overeating	Negative affect may reduce food approach and promote eating avoidance	EE should not be treated as a single overeating phenotype
Reward-motivational processing	Heightened food-cue salience and reward-driven engagement	Attenuated reward anticipation, evaluation, or feedback	EOE may reflect reward amplification, whereas EUE may reflect reward attenuation
Attentional allocation to food cues	Enhanced early and sustained attention to food cues	Limited direct evidence; possible reduced salience of appetitive cues	Food cues may pull attention more strongly in EOE than in EUE
Cognitive control and conflict monitoring	Insufficient conflict detection and inhibitory-control recruitment	Heightened or dysregulated monitoring with inefficient adjustment	EOE may involve under-recruitment of control, whereas EUE may involve over-monitoring without efficient regulation
Functional imbalance pathway	Reward amplification with insufficient control	Reward attenuation with excessive monitoring	The two phenotypes may reflect divergent imbalance pathways rather than being opposite ends of one continuum
Exercise-related hypothesis	Exercise may strengthen control and reduce excessive food-cue salience	Exercise may support reward responsiveness and attenuate excessive monitoring	Exercise effects may be baseline-dependent and phenotype-specific
Evidence boundary	Mainly indirect ERP evidence from food cues, BED, obesity, high EE, or non-depressive samples	Mainly indirect/inferential evidence from depression, anhedonia, reduced appetite, reward processing, and error monitoring studies	Comparative conclusions remain hypothesis-generating and require phenotype-stratified ERP trials

SPN, stimulus-preceding negativity; ERN, error-related negativity; EUE, emotional undereating; EOE, emotional overeating; LPP, late positive potential; ERP, event-related potential; RewP, reward positivity; Pe, error positivity. Note. Comparative profiles are mainly based on indirect and inferential evidence. EOE and EUE are unevenly supported across ERP components, paradigms, and stimulus contexts, with no direct ERP comparisons in depressive populations. Thus, this table presents a component-specific, hypothesis-generating synthesis rather than a validated model. EUE-related conclusions should not be equated with appetite loss or anhedonia.

## 4 ERP-informed hypotheses on exercise modulation of neural mechanisms related to EOE and EUE

### 4.1 ERP evidence for exercise modulation of neural mechanisms underlying EOE

In depressive populations, the core mechanism underlying EOE has been hypothesized to involve weakened top-down cognitive control mediated by the PFC and ACC, accompanied by enhanced bottom-up attentional processing of food cues (73, 74). ERP studies have indicated that exercise interventions do not exert their effects through a single neural locus. Instead, exercise may contribute to a partial recalibration of this functional imbalance by modulating multiple stages of neural processing, ranging from conflict monitoring and inhibitory execution to emotional processing (36, 75–80).

At the level of cognitive control, existing studies have indicated that regular aerobic exercise may modulate N2 and P3 amplitudes during general cognitive tasks in individuals with depression, potentially reflecting improved executive control engagement (32). However, in food-related inhibitory tasks, the regulatory effects of a single bout of moderate-intensity aerobic exercise appear to be relatively limited. Evidence from food-related ERP studies, obesity samples, and review-level synthesis has suggested that, although moderate-intensity exercise can improve general cognitive processing, it cannot robustly enhance N2 and P3 amplitudes in the presence of high-calorie food cues (77, 79, 81, 82). This pattern suggests that the cognitive resources mobilized by moderate-intensity exercise remain insufficient to counteract strong food-related motivational processing. In contrast, high-intensity interval training (HIIT) and resistance exercise have demonstrated more consistent effects in modulating food-related inhibitory control. HIIT has been associated with improvements in executive function and may modulate inhibitory control-related ERP indices, including P3. However, direct evidence from food-related inhibitory tasks in depressive EOE-stratified samples remains limited (31, 82). Resistance exercise has been associated with shorter P3 latencies (83), suggesting more efficient cognitive processing and executive control recruitment. These findings provide indirect support for the hypothesis that exercise may improve inhibitory-control processes relevant to EOE, but they should not be interpreted as direct evidence of reduced eating impulses in depressive EOE populations. During stages of sustained attentional allocation and emotional processing, changes in LPP further reveal the task-dependent nature of exercise modulation. Among individuals who engage in long-term regular exercise, there are reduced LPP amplitudes during passive viewing of food images, which reflects decreased motivational salience of food cues and attenuated desire responses at the neural level (34). Conversely, during tasks that require active inhibition or emotion regulation, high-intensity exercise may enhance LPP amplitudes, reflecting the further recruitment of PFC resources for attentional and emotional control (84). The acute effects and long-term adaptations should be distinguished in exercise interventions. Some studies have suggested that high-dose acute aerobic exercise may transiently increase attentional bias toward food cues via metabolic compensation or heightened motivation, thereby weakening gains in cognitive control (85, 86). These effects are less frequently observed after resistance training, HIIT, or long-term regular exercise, possibly owing to their sustained activation of PFC executive function (31, 87, 88). Accordingly, HIIT, resistance training, and long-term regular exercise may be considered promising areas for future EOE-focused intervention studies, but they should not be interpreted as established intervention prescriptions. A stratified overview of ERP-informed evidence relevant to potential exercise modulation of depression-related EOE mechanisms is presented in Table 6.

**Table 6:** Stratified evidence for the potential exercise modulation of EOE-related neural mechanisms.

Processing stage	ERP	Exercise modality	Primary neurocognitive interpretation	Evidence level	References
Early conflict monitoring	N2 ↑	Regular aerobic exercise	Potential enhancement of conflict monitoring and executive control engagement	Indirect evidence	(32)
	N2 (no significant change)	Single bout of moderate-intensity aerobic exercise	Limited modulation of food-related motivational interference	Indirect and inferential evidence	(77, 79, 81, 82)
Inhibitory	P3 ↑ or P3 modulation	Single bout of moderate-intensity aerobic exercise	Limited improvement in food-related inhibitory control allocation under high-calorie food-cue conditions	Indirect and inferential evidence	(77, 79, 81, 82)
	P3 ↑	HIIT	Potential increase in inhibitory control resource allocation	Indirect and inferential evidence	(99, 100)
	P3 Latency ↓	Resistance exercise	Potential acceleration of cognitive processing and executive control efficiency	Indirect evidence	(83)
Sustained attention and emotional processing	LPP ↓	Long-term regular exercise	Potential reduction in motivational salience of food cues and craving-related responses	Indirect evidence	(34)
	LPP ↑	High-intensity exercise	Possible task-dependent recruitment of attentional or emotional regulation resources	Indirect and inferential evidence	(84)
Temporal effects	—	High-dose acute aerobic exercise	Possible transient increase in attentional bias or food motivation through compensatory mechanisms	Indirect and inferential evidence	(85, 86)
	—	HIIT, resistance training, and long-term exercise	Hypothesis-generating support for executive control improvement; EOE-specific ERP evidence remains limited	Inferential evidence	(31, 87)
	—	Acute high-intensity interval exercise	Possible attenuation of high-fat food craving incubation	Inferential evidence	(88)

HIIT, high-intensity interval training; EOE, emotional overeating; LPP, late positive potential; ERP, event-related potential; ↑, increase; ↓, decrease. No studies included in this table meet the criteria for direct evidence of exercise-related ERP modulation in depressive EOE populations. Evidence is therefore classified as indirect or inferential according to population relevance, phenotype specificity, task relevance, and study design. Exercise-related implications should be interpreted as hypothesis-generating rather than prescriptive recommendations.

### 4.2 ERP evidence for exercise modulation of neural mechanisms underlying EUE

Unlike EOE, the putative EUE phenotype in depression may be associated with attenuated reward-motivational processing accompanied by abnormalities in conflict-monitoring systems. Thus, the hypothesized neural regulatory effects of exercise on EUE may not simply involve strengthening inhibitory control but may instead involve partial recalibration of reward processing and conflict monitoring mechanisms. Exercise could theoretically support functional connectivity between the PFC and striatum and may increase engagement of the anterior insula and vmPFC during reward anticipation, thereby ameliorating insufficient motivational mobilization (89). Exercise may theoretically modulate ACC-related functional states, potentially attenuating excessive error sensitivity and supporting more efficient neural processing; however, this pathway requires direct validation in EUE-stratified ERP intervention studies (90). Consequently, exercise type, intensity, and duration represent important variables to examine in future EUE-focused intervention studies.

During the reward processing stage, the available evidence suggests that regular moderate-intensity aerobic exercise may be a promising direction for future EUE-focused intervention studies, rather than an established intervention prescription (91). Mechanistically, such an exercise may be associated with anterior insula engagement during reward anticipation (92, 93) and may provide a possible pathway for addressing SPN-related reward anticipation attenuation inferred for putative EUE-related profiles (59), and for supporting sensitivity to reward-related cues (94). Several interventions lasting approximately 8 weeks have shown that moderate-intensity aerobic exercise can improve overall reward system functioning and exert modulatory effects on RewP. However, direct evidence for sustained and robust enhancement of RewP remains limited (91, 95). Although there has been very little direct examination of eating-related RewP in EUE-stratified samples, the generality of reward circuitry suggests that these effects could transfer to eating-related contexts. By comparison, ERP evidence for resistance training in reward processing remains limited. Nevertheless, animal studies have suggested that resistance exercise may influence reward processing via modulation of dopaminergic function in the nucleus accumbens (96). Overall, exercise may be hypothesized to support shifts in reward-related neural activity toward a more adaptive functional range in individuals with putative EUE-related profiles.

At the stages of conflict monitoring and behavioral adjustment, indirect and inferential evidence from studies involving depression-related cognitive control abnormalities and avoidance-related processing has suggested that putative EUE-related profiles are associated with increased N2 and ERN amplitudes alongside reduced Pe. This pattern may reflect heightened monitoring of negative information and potentially inefficient recruitment of cognitive-control resources. Notably, elevated N2 and ERN do not indicate more efficient cognitive control. Instead, these suggest that the ACC operates under a persistently high load and is unable to achieve effective regulation through subsequent processing stages. Exercise-based interventions may help optimize neural resource allocation at this stage and may promote more adaptive conflict-monitoring function. Existing studies have indicated that aerobic exercise can modulate ERN and Pe in depressive populations, although the findings are inconsistent (95). Notably, some randomized controlled trials have reported that moderate-intensity aerobic exercise has been associated with increased N2 amplitude, signifying an improved capacity for effective resource recruitment and improved conflict processing efficiency (33, 97). Additionally, aerobic exercise combined with meditation training, specifically mindfulness awareness practices (MAPs), has been shown to simultaneously increase N2 and P3 amplitudes, suggesting improved conflict monitoring and behavioral adjustment capacity (98). In contrast, although HIIT is able to activate PFC function, its effects on ERP components related to conflict monitoring lack consistent evidence and are derived predominantly from studies that were conducted among healthy individuals (33, 77, 99–101).

The available evidence suggests that regular moderate-intensity aerobic exercise, potentially with individualized progression in intensity, may help shift functional activity within the PFC–striatal–ACC networks toward a more balanced range. Through this hypothesized mechanism, individuals who exhibit EUE may show improved reward motivation and reduced excessive conflict monitoring, although this possibility requires direct validation. However, it should be emphasized that direct ERP studies examining the effects of exercise specifically in EUE populations are limited and that these conclusions require further empirical validation. A stratified overview of ERP evidence for exercise modulation of EUE in depressive populations is presented in Table 7.

**Table 7:** Stratified evidence for the potential exercise modulation of EUE-related neural mechanisms.

Processing stage	ERP	Exercise modality	Primary neurocognitive interpretation	Evidence level	References
Reward anticipation	SPN ↑	Moderate- to high-intensity aerobic exercise	Potential enhancement of reward prediction and anticipatory motivation	Indirect and inferential evidence	(59, 91–94)
Reward feedback	RewP (↑ or no significant change)	Moderate-intensity aerobic exercise lasting at least 8 weeks	Possible improvement in reward-feedback processing, although effects remain inconsistent	Indirect evidence	(91, 95)
Reward processing	—	Resistance training	Potential modulation of dopaminergic pathways	Inferential evidence	(96)
Conflict monitoring	N2 ↑, P3 ↑	Moderate-intensity aerobic exercise, MAPs	Potential improvement in conflict monitoring and cognitive-control allocation	Indirect and inferential evidence	(33, 97, 98)
Error monitoring	ERN ↓/Pe ↑	Aerobic exercise	Possible reduction in excessive monitoring and improvement in post-error adjustment	Indirect evidence	(95)
Executive control	N2 ↑/ERN (no significant change)	HIIT	Potential activation of executive-control networks	Inferential evidence	(33, 77, 99–101)
Integrated regulation	—	Regular moderate-intensity aerobic exercise	Hypothesis-generating evidence for potential support of reward-related functioning and attenuation of excessive monitoring	Inferential evidence	(89–91, 95, 97)

HIIT, high-intensity interval training; SPN, stimulus-preceding negativity; ERN, error-related negativity; EUE, emotional undereating; LPP, late positive potential; ERP, event-related potential; RewP, reward positivity; Pe, error positivity; MAPs, Mindfulness awareness practices; ↑, increase; ↓, decrease. No studies included in this table meet the criteria for direct evidence of exercise-related ERP modulation in depressive EUE populations. Evidence was therefore classified as indirect or inferential according to population relevance, phenotype specificity, task relevance, and study design. Integrative statements were treated as a subtype of inferential evidence and should be interpreted as hypothesis-generating rather than as empirical confirmation of EUE-specific exercise effects.

### 4.3 A proposed shared pathway through which exercise may modulate EOE and EUE

**Figure 3:**
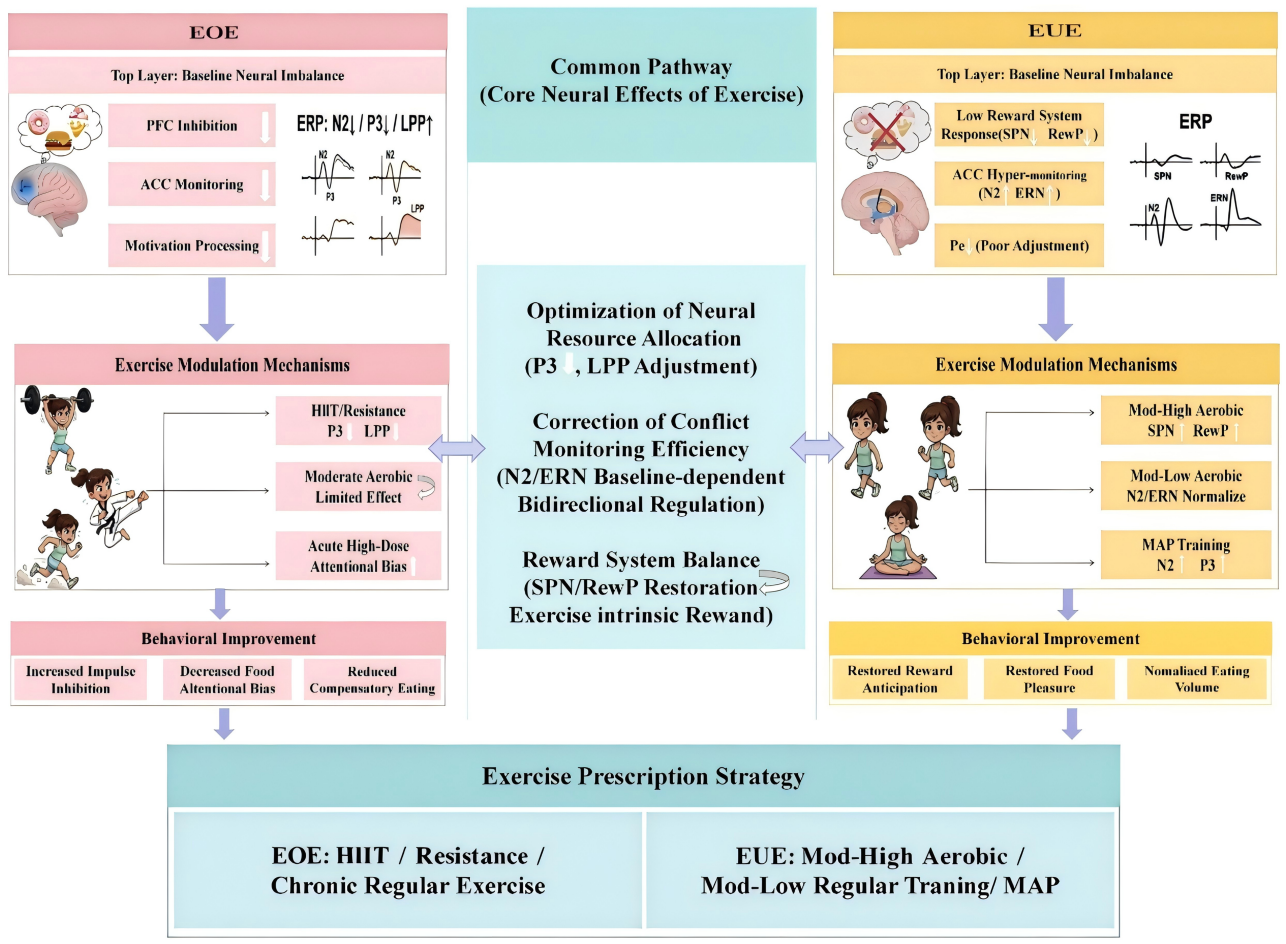
A hypothesis-generating, bidirectional regulatory framework of the potential effects of exercise on EoE and EUE via a shared neural pathway.

Although EOE and EUE manifest as opposite eating behaviors, current evidence does not support two fully independent or directly validated exercise-regulatory mechanisms for these phenotypes. Instead, based on the available direct, indirect, and inferential evidence, we propose a shared and baseline-dependent pathway through which exercise may differentially modulate EOE and EUE (Figure 3). This conceptual pathway centers on exercise-related changes in PFC and ACC-mediated cognitive control networks, which may influence reward system responsivity and thereby shift motivational processing and behavioral control toward a more functionally balanced range (102, 103).

In this pathway, the PFC and ACC support attentional resource allocation, conflict monitoring, and the maintenance of behavioral goals. Exercise may support more efficient engagement of this network, thereby facilitating the allocation of neural resources to goal-directed processing and reducing excessive engagement with emotional or reward-related cues. At the ERP level, such a possibility may be reflected as changes in P3 and LPP amplitudes, although these indices remain task-dependent and cannot be interpreted as process-pure markers (31, 82, 104).

Through its possible effects on cognitive-control networks, exercise could contribute to the modulation of reward-system responsiveness. Exercise has been proposed to act as an endogenous reward-related stimulus and to engage dopamine-related pathways without relying exclusively on food cues (105). For individuals with exaggerated food-reward responses, as hypothesized for EOE, exercise may reduce the motivational salience of food cues, which could be reflected in lower food-related LPP amplitudes (35, 106, 107). For individuals with blunted reward responses, such as those hypothesized to characterize EUE, exercise may support reward anticipation and feedback processing, potentially involving SPN and RewP modulation (59, 91, 95, 107, 108). These pathways should be interpreted as hypothesis-generating predictions rather than as directly validated ERP effects in phenotype-stratified depressive samples.

Concurrently, exercise may exert baseline-dependent modulation of the conflict-monitoring system. By potentially modulating ACC-related functional states, exercise may help adjust abnormally low or excessively high conflict-monitoring activity toward a more efficient range, preserving necessary conflict detection while preventing excessive vigilance-induced behavioral inhibition (32, 33, 101).

In summary, we propose a shared, baseline-dependent regulatory pathway through which exercise may differentially modulate EOE and EUE. This pathway is centered on cognitive-control networks involving the PFC and ACC and may support more adaptive neural resource allocation, coordination between conflict monitoring and reward responsiveness, and functional balance between cognitive control and reward-driven processes. Importantly, this pathway should be interpreted as a conceptual synthesis based on stratified evidence rather than as a directly validated neural mechanism. It therefore provides a hypothesis-generating framework for future studies examining whether baseline neurocognitive characteristics can inform more precise exercise interventions for depression-related emotional eating.

## 5 Construction of a bidirectional neurocognitive model of exercise regulation

**Figure 4:**
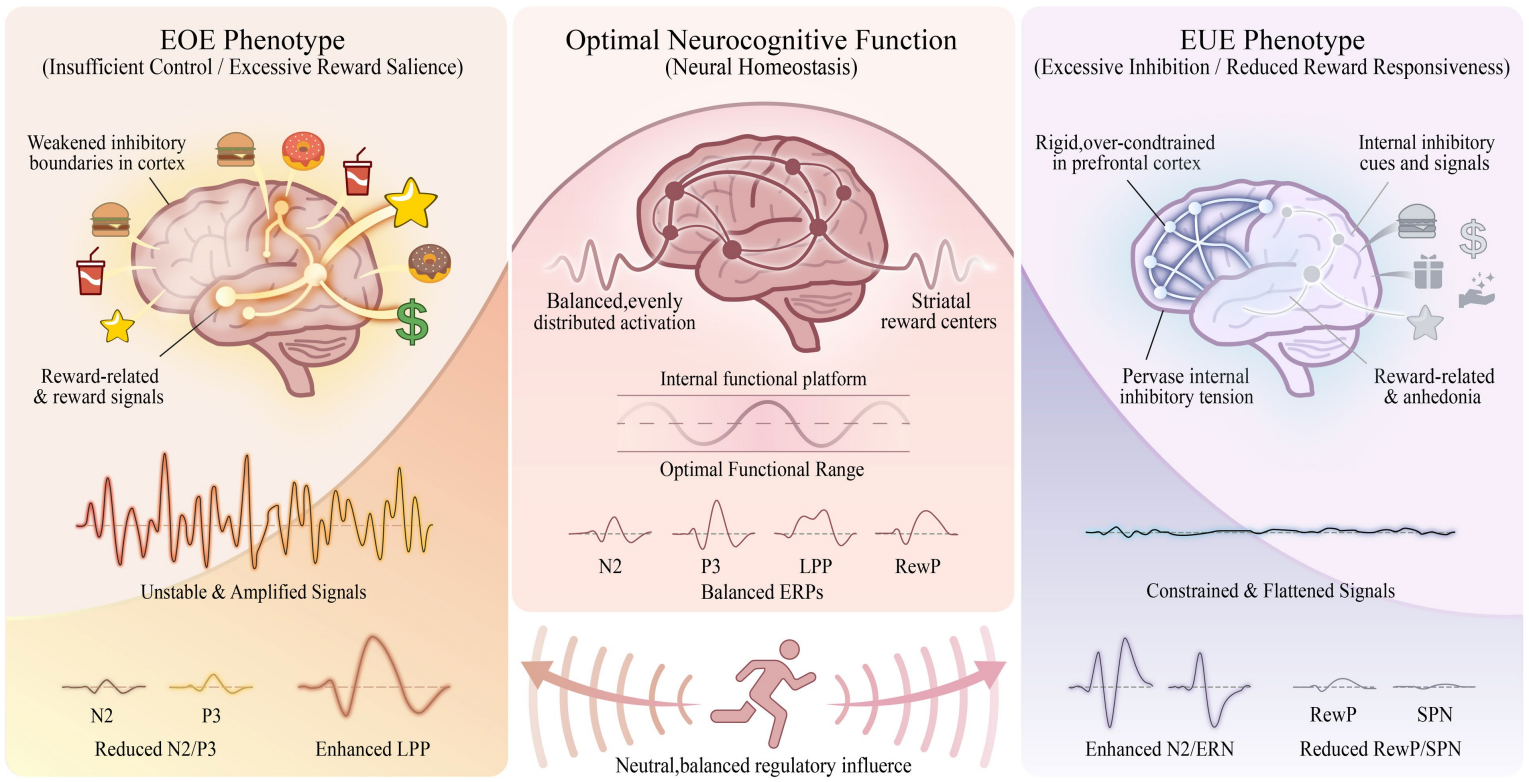
A conceptual, bidirectional neurocognitive model of exercise regulation based on putative neural homeostasis mechanisms.

In exercise psychology and clinical intervention research, the effects of exercise are often examined through a linear dose–response framework, in which exercise-related modulation of neurocognitive function is treated as a homogeneous enhancement effect. However, the ERP evidence summarized above reveals pronounced heterogeneity in eating behavior under depressive conditions. Specifically, EOE and EUE may exhibit directionally divergent patterns of neural imbalance at the electrophysiological level (22, 43). These putative mechanistic differences suggest that traditional unidirectional intervention perspectives may be insufficient to capture the complexity of the effects of exercise. Accordingly, we propose a bidirectional intervention model in which exercise is hypothesized to function as a regulator of neural homeostasis (Figure 4). The central premise of this model is that exercise may not mechanically enhance or suppress activity within specific brain regions but may instead operate as a dynamic regulatory process dependent on an individual’s baseline neural state (1, 56). Exercise intervention may contribute to shifts in neurocognitive processing toward a more functionally balanced range, potentially through modulation of systems hypothesized to deviate from adaptive functional ranges, thereby providing a possible basis for bidirectional rather than unidirectional regulatory effects. Based on characteristic patterns derived from ERP evidence, the proposed model comprises two core regulatory pathways: cognitive control and reward motivation. The putative imbalances expressed within these pathways may provide a theoretical basis for future studies of phenotype-informed exercise modulation.

This model represents a conceptual synthesis based on stratified evidence and should not be interpreted as a directly validated phenotype-specific mechanism.

Within the cognitive control pathway, EOE and EUE are characterized by opposing patterns of dysregulation, namely, insufficient inhibition and excessive inhibition. Individuals who exhibit EOE display attenuated N2 and No-Go P3 responses when exposed to highly tempting food cues, indicating inadequate recruitment of PFC resources and impaired inhibitory control. This pattern is consistently observed across both general inhibition tasks and food-related paradigms (40, 44), and it is closely associated with impulsive eating behavior and failures in emotion regulation (46). In individuals with putative EOE-related profiles, exercise may hypothetically support inhibitory-control recruitment, potentially through neuroplastic changes involving PFC and ACC circuits (29, 37). Conversely, putative EUE-related profiles may not simply exhibit deficient control; rather, they may instead show heightened or dysregulated monitoring, as inferred from depression-related conflict- and error-monitoring evidence. This pattern may be indexed by enhanced N2 or ERN responses and may be consistent with elevated ACC-related monitoring demands rather than a directly validated EUE-specific mechanism (25, 67). This putative inhibitory tendency may consume the cognitive resources required for physiologically driven eating and may contribute to eating avoidance, particularly when accompanied by anhedonia-related reward blunting (13). In response to this putative profile, exercise may theoretically attenuate excessive ACC-related monitoring by alleviating negative affect and modulating stress-related systems, thereby supporting a more adaptive allocation of cognitive resources (38).

Within the reward motivation pathway, EOE and EUE correspond to the two opposing extremes of reward sensitization and anhedonia. Among individuals with EOE, enhanced LPP responses indicate the excessive motivational salience of food cues and are closely linked to striatal hyper-responsivity and a negative reinforcement cycle of EE (22, 41, 109). This pattern is consistent with exaggerated striatal reactivity and the maintenance of EE via negative reinforcement mechanisms (41). As an endogenous reward-related stimulus, exercise may theoretically provide an alternative source of reward engagement and shift reward salience away from food-related cues. In long-term training, exercise may be associated with reduced food cue salience within reward-related processing, as indexed by decreased food-related LPP amplitudes (35). In contrast, individuals with EUE display convergent attenuation across the SPN, P3, and RewP components, indicating a hypo-functional reward system (59, 60). Under these conditions, exercise may support dopaminergic activity and baseline reward system functioning, potentially contributing to improved motivational responsiveness to food cues (55).

At an integrative level, the two pathways discussed above converge into a neural homeostasis model that follows an inverted U-shaped principle. This model posits that neurocognitive function operates optimally within a moderate activation range, with EOE and EUE occupying opposite deviations from this functional zone. Exercise may contribute to the reallocation of cognitive resources and optimization of neural efficiency, potentially by shifting hypoactive or hyperactive systems toward a more balanced range and by supporting functional coordination between cognitive control and reward-driven processes. This model marks a shift in EE interventions from uniform strategies to a precision-based paradigm. In future clinical research, behavioral assessments could be combined with neural indicators to distinguish EE phenotypes and inform the development of differentiated exercise-intervention hypotheses. Future research should include randomized controlled trials to further test the specific predictions of this model and elucidate the micro-level mechanisms through which electrophysiological changes translate into behavioral improvement, thereby advancing neurobiologically informed, personalized exercise interventions. More broadly, the present model is intended to organize currently fragmented evidence and generate testable predictions, rather than to provide a confirmed explanatory account of exercise effects on depression-related emotional eating.

## 6 Limitations and future outlook

### 6.1 Limitations

Although accumulating evidence supports the existence of two opposing EE phenotypes in depression-related EE-EOE and EUE, few studies have directly examined depressive groups and systematically assessed ERP indices using emotion-induced and food-related tasks (19, 20). Thus, several conclusions necessarily rely on mechanistic extrapolation across populations and task paradigms. EE research has also relied heavily on self-report questionnaires (16–18), which have a limited ability to capture the dynamic neural processing triggered by negative emotions and often reduce EE to excessive eating, thereby hindering recognition of the EUE phenotype (11).

Changes in appetite or body weight, included in the diagnostic criteria for depression, are not equivalent to EE at either the conceptual or the mechanistic level. Failure to distinguish between these constructs may confound neural interpretations and obscure intervention targets (2, 3, 13–15). From a methodological perspective, substantial heterogeneity exists across studies with respect to emotion induction procedures, task paradigms, and ERP component selection, which constrains comparability and the integrative synthesis of findings (21–24, 26, 27). Within intervention research, although exercise has been consistently shown to alleviate depressive symptoms (29, 30), electrophysiological evidence stratified according to the EOE and EUE phenotypes remains scarce. This limitation prevents the direct testing of specific predictions derived from the proposed bidirectional regulatory model (35, 37, 38). Relatedly, although the evidence-tier framework has helped reduce overinterpretation across populations, tasks, and evidence types, it should not be interpreted as a formal quality appraisal or risk-of-bias assessment. The direct, indirect, and inferential categories indicate inferential proximity to the proposed model rather than the methodological quality of individual studies.

### 6.2 Future outlook

Future research should prioritize the establishment of a unified framework for identifying EE phenotypes that integrates self-report measures, behavioral tasks, and ERP indices. Such an approach would allow for clear differentiation between EOE and EUE, and delineate their boundaries from depression-related changes in appetite or body weight (16–20). On this basis, systematic ERP studies among depressive groups that combine emotion induction with food cue paradigms are needed. These studies should encompass key processing stages, including attention, conflict monitoring, inhibitory execution, and reward processing, to empirically test and refine the proposed bidirectional neural imbalance model (21–24, 26, 27). At the intervention level, phenotype-stratified randomized controlled exercise trials should be conducted to compare various exercise modalities and intensities, while explicitly distinguishing between acute effects and long-term adaptations. With such a design, it could be evaluated whether exercise produces differential neural regulatory effects, as predicted by the model (29, 30, 35, 37, 38). Further integration of stress- and reward-related indicators could facilitate the construction of mechanistic pathways that link electrophysiological changes to improvements in eating behavior, thereby providing a foundation for neurobiologically informed, individualized exercise interventions.

## 7 Conclusion

This review highlights pronounced neural heterogeneity in depression-related EE. EOE may involve reward sensitization combined with weakened inhibitory control, whereas EUE may involve attenuated reward processing combined with excessive conflict or error monitoring. Based on stratified evidence with varying inferential distances, we propose a homeostasis-oriented conceptual framework in which exercise is hypothesized to modulate depression-related EE in a baseline-dependent manner. For EOE, exercise is hypothesized to support PFC-mediated control processes and attenuate the motivational salience of food cues. For EUE, exercise is hypothesized to potentially support reward responsiveness and attenuate excessive monitoring. However, these pathways remain theoretical and require direct validation in phenotype-stratified ERP intervention studies. Future research should integrate phenotype-based assessments, emotion-induced food-related ERP paradigms, and multimodal neural indicators to test and refine this model.
