# Supplementary material for: Bidirectional regulatory effects of exercise on emotional eating in depression: an ERP-based narrative review
Source: Front Nutr. 2026 May 29;13:1832858. doi: 10.3389/fnut.2026.1832858 (PMC13259850; doi:10.3389/fnut.2026.1832858)
Supplement: Supplementary file 1 [file Table_1.DOC]

**Supplementary Appendix 1** Search Strategy 2026.2.17

Supplementary Appendix 1 presents the database-specific search strategies used in this narrative review. Because the review aimed to construct an ERP-informed conceptual model across depression, emotional eating phenotypes, food-cue processing, and exercise-related neural modulation, the search was organized into five thematic blocks rather than a single restrictive search string. Blocks 1–4 were used to identify evidence relevant to different components of the model, including depression-related emotional eating phenotypes, food-cue ERP processing, depression-related reward and control mechanisms, and exercise-related ERP modulation. Block 5 was used as a supplementary comprehensive search to identify studies that directly combined depression, emotional eating or food-cue processing, ERP/EEG indices, and exercise. The number of records refers to raw database results before deduplication, and records across search blocks may overlap.

| **Search Query** | **Search strategy** | **Records retrieved** |
| --- | --- | --- |
| **PubMed** | | |
| #1  Depression and Emotional Eating Phenotype | **("Depression"[MeSH] OR "Depressive Disorder"[MeSH] OR depression[Text Word] OR "depressive disorder"[Text Word] OR "major depressive disorder"[Text Word] OR "depressive symptoms"[Text Word] OR anhedonia[Text Word])**  **AND**  **("emotional eating"[Text Word] OR "emotional overeating"[Text Word] OR "emotional over-eating"[Text Word] OR "emotional undereating"[Text Word] OR "emotional under-eating"[Text Word] OR overeating[Text Word] OR "over-eating"[Text Word] OR undereating[Text Word] OR "under-eating"[Text Word] OR "appetite change"[Text Word] OR "increased appetite"[Text Word] OR "decreased appetite"[Text Word] OR "appetite loss"[Text Word] OR "eating behavior"[Text Word])** | 2,002 results |
| #2  Food Cues, Emotional Eating, and ERP | **("emotional eating"[Text Word] OR "binge eating"[Text Word] OR "binge eating disorder"[Text Word] OR obesity[Text Word] OR overweight[Text Word] OR "food craving"[Text Word] OR "food cue"[Text Word] OR "food cues"[Text Word] OR "food stimuli"[Text Word] OR "high-calorie food"[Text Word] OR "palatable food"[Text Word])**  **AND**  **("event-related potential"[Text Word] OR "event-related potentials"[Text Word] OR ERP[Text Word] OR EEG[Text Word] OR P1[Text Word] OR N1[Text Word] OR N2[Text Word] OR P3[Text Word] OR P300[Text Word] OR LPP[Text Word] OR "late positive potential"[Text Word] OR SPN[Text Word] OR "stimulus-preceding negativity"[Text Word] OR RewP[Text Word] OR "reward positivity"[Text Word] OR ERN[Text Word] OR "error-related negativity"[Text Word] OR Pe[Text Word] OR "error positivity"[Text Word])** | 2,331 results |
| #3  Depression, Reward Processing, Conflict Monitoring, and ERP | **("Depression"[MeSH] OR "Depressive Disorder"[MeSH] OR depression[Text Word] OR "depressive disorder"[Text Word] OR "major depressive disorder"[Text Word] OR "depressive symptoms"[Text Word] OR anhedonia[Text Word])**  **AND**  **("reward processing"[Text Word] OR "reward responsiveness"[Text Word] OR "reward sensitivity"[Text Word] OR "reward anticipation"[Text Word] OR "reward feedback"[Text Word] OR "conflict monitoring"[Text Word] OR "error monitoring"[Text Word] OR "inhibitory control"[Text Word] OR "cognitive control"[Text Word])**  **AND**  **("event-related potential"[Text Word] OR "event-related potentials"[Text Word] OR ERP[Text Word] OR EEG[Text Word] OR N2[Text Word] OR P3[Text Word] OR P300[Text Word] OR LPP[Text Word] OR "late positive potential"[Text Word] OR SPN[Text Word] OR "stimulus-preceding negativity"[Text Word] OR RewP[Text Word] OR "reward positivity"[Text Word] OR ERN[Text Word] OR "error-related negativity"[Text Word] OR Pe[Text Word] OR "error positivity"[Text Word])** | 347 results |
| #4  Exercise, Depression/Eating Behavior, and ERP | **("Exercise"[MeSH] OR "Motor Activity"[MeSH] OR exercise[Text Word] OR "physical activity"[Text Word] OR "aerobic exercise"[Text Word] OR "resistance exercise"[Text Word] OR "high-intensity interval training"[Text Word] OR HIIT[Text Word] OR "acute exercise"[Text Word] OR "exercise intervention"[Text Word] OR "exercise training"[Text Word])**  **AND**  **("Depression"[MeSH] OR "Depressive Disorder"[MeSH] OR depression[Text Word] OR "depressive symptoms"[Text Word] OR "major depressive disorder"[Text Word] OR "emotional eating"[Text Word] OR "food cue"[Text Word] OR "food cues"[Text Word] OR "food craving"[Text Word] OR appetite[Text Word] OR "eating behavior"[Text Word])**  **AND**  **("event-related potential"[Text Word] OR "event-related potentials"[Text Word] OR ERP[Text Word] OR EEG[Text Word] OR N2[Text Word] OR P3[Text Word] OR P300[Text Word] OR LPP[Text Word] OR "late positive potential"[Text Word] OR SPN[Text Word] OR "stimulus-preceding negativity"[Text Word] OR RewP[Text Word] OR "reward positivity"[Text Word] OR ERN[Text Word] OR "error-related negativity"[Text Word] OR Pe[Text Word] OR "error positivity"[Text Word])** | 292 results |
| #5  Direct-evidence search combining depression, emotional eating/food cues, ERP/EEG, and exercise | **("Depression"[MeSH] OR "Depressive Disorder"[MeSH] OR depression[Text Word] OR "depressive disorder"[Text Word] OR "major depressive disorder"[Text Word] OR "depressive symptoms"[Text Word] OR anhedonia[Text Word]) AND ("emotional eating"[Text Word] OR "emotional overeating"[Text Word] OR "emotional over-eating"[Text Word] OR "emotional undereating"[Text Word] OR "emotional under-eating"[Text Word] OR overeating[Text Word] OR undereating[Text Word] OR "appetite change"[Text Word] OR "food cue"[Text Word] OR "food cues"[Text Word] OR "food craving"[Text Word] OR "eating behavior"[Text Word]) AND ("event-related potential"[Text Word] OR "event-related potentials"[Text Word] OR ERP[Text Word] OR EEG[Text Word] OR P1[Text Word] OR N1[Text Word] OR N2[Text Word] OR P3[Text Word] OR P300[Text Word] OR LPP[Text Word] OR "late positive potential"[Text Word] OR SPN[Text Word] OR "stimulus-preceding negativity"[Text Word] OR RewP[Text Word] OR "reward positivity"[Text Word] OR ERN[Text Word] OR "error-related negativity"[Text Word] OR Pe[Text Word] OR "error positivity"[Text Word]) AND ("Exercise"[MeSH] OR "Motor Activity"[MeSH] OR exercise[Text Word] OR "physical activity"[Text Word] OR "aerobic exercise"[Text Word] OR "resistance exercise"[Text Word] OR "high-intensity interval training"[Text Word] OR HIIT[Text Word] OR "exercise intervention"[Text Word] OR "exercise training"[Text Word])** | 3 results |

| **Search Query** | **Search strategy** | **amount** |
| --- | --- | --- |
| **Web of Science** | | |
| #1  Depression and Emotional Eating Phenotype | **TS = ( (depression OR "depressive disorder" OR "major depressive disorder" OR "depressive symptoms" OR anhedonia) AND ("emotional eating" OR "emotional overeating" OR "emotional over-eating" OR "emotional undereating" OR "emotional under-eating" OR overeating OR "over-eating" OR undereating OR "under-eating" OR "appetite change" OR "increased appetite" OR "decreased appetite" OR "appetite loss" OR "eating behavior") )** | 3,938 results |
| #2  Food Cues, Emotional Eating, and ERP | **TS = ( ("emotional eating" OR "binge eating" OR "binge eating disorder" OR obesity OR overweight OR "food craving" OR "food cue" OR "food cues" OR "food stimuli" OR "high-calorie food" OR "palatable food") AND ("event-related potential" OR "event-related potentials" OR ERP OR EEG OR P1 OR N1 OR N2 OR P3 OR P300 OR LPP OR "late positive potential" OR SPN OR "stimulus-preceding negativity" OR RewP OR "reward positivity" OR ERN OR "error-related negativity" OR Pe OR "error positivity") )** | 2,602 results |
| #3  Depression, Reward Processing, Conflict Monitoring, and ERP | **TS = ( (depression OR "depressive disorder" OR "major depressive disorder" OR "depressive symptoms" OR anhedonia) AND ("reward processing" OR "reward responsiveness" OR "reward sensitivity" OR "reward anticipation" OR "reward feedback" OR "conflict monitoring" OR "error monitoring" OR "inhibitory control" OR "cognitive control") AND ("event-related potential" OR "event-related potentials" OR ERP OR EEG OR N2 OR P3 OR P300 OR LPP OR "late positive potential" OR SPN OR "stimulus-preceding negativity" OR RewP OR "reward positivity" OR ERN OR "error-related negativity" OR Pe OR "error positivity") )** | 677 results |
| #4  Exercise, Depression/Eating Behavior, and ERP | **TS = ( (exercise OR "physical activity" OR "aerobic exercise" OR "resistance exercise" OR "high-intensity interval training" OR HIIT OR "acute exercise" OR "exercise intervention" OR "exercise training") AND (depression OR "depressive symptoms" OR "major depressive disorder" OR "emotional eating" OR "food cue" OR "food cues" OR "food craving" OR appetite OR "eating behavior") AND ("event-related potential" OR "event-related potentials" OR ERP OR EEG OR N2 OR P3 OR P300 OR LPP OR "late positive potential" OR SPN OR "stimulus-preceding negativity" OR RewP OR "reward positivity" OR ERN OR "error-related negativity" OR Pe OR "error positivity") )** | 298 results |
| #5  Direct-evidence search combining depression, emotional eating/food cues, ERP/EEG, and exercise | **TS = ( (depression OR "depressive disorder" OR "major depressive disorder" OR "depressive symptoms" OR anhedonia) AND ("emotional eating" OR "emotional overeating" OR "emotional over-eating" OR "emotional undereating" OR "emotional under-eating" OR overeating OR undereating OR "appetite change" OR "food cue" OR "food cues" OR "food craving" OR "eating behavior") AND ("event-related potential" OR "event-related potentials" OR ERP OR EEG OR P1 OR N1 OR N2 OR P3 OR P300 OR LPP OR "late positive potential" OR SPN OR "stimulus-preceding negativity" OR RewP OR "reward positivity" OR ERN OR "error-related negativity" OR Pe OR "error positivity") AND (exercise OR "physical activity" OR "aerobic exercise" OR "resistance exercise" OR "high-intensity interval training" OR HIIT OR "exercise intervention" OR "exercise training") )** | 4 results |

| **Search Query** | **Search strategy** | **amount** |
| --- | --- | --- |
| **Embase** | | |
| #1  Depression and Emotional Eating Phenotype | **('depression'/exp OR 'depressive disorder'/exp OR depression:ti,ab,kw OR 'depressive disorder':ti,ab,kw OR 'major depressive disorder':ti,ab,kw OR 'depressive symptoms':ti,ab,kw OR anhedonia:ti,ab,kw) AND ('emotional eating':ti,ab,kw OR 'emotional overeating':ti,ab,kw OR 'emotional over-eating':ti,ab,kw OR 'emotional undereating':ti,ab,kw OR 'emotional under-eating':ti,ab,kw OR overeating:ti,ab,kw OR 'over-eating':ti,ab,kw OR undereating:ti,ab,kw OR 'under-eating':ti,ab,kw OR 'appetite change':ti,ab,kw OR 'increased appetite':ti,ab,kw OR 'decreased appetite':ti,ab,kw OR 'appetite loss':ti,ab,kw OR 'eating behavior':ti,ab,kw)** | 3,970 results |
| #2  Food Cues, Emotional Eating, and ERP | **('emotional eating':ti,ab,kw OR 'binge eating'/exp OR 'binge eating':ti,ab,kw OR 'binge eating disorder'/exp OR 'binge eating disorder':ti,ab,kw OR obesity/exp OR obesity:ti,ab,kw OR overweight/exp OR overweight:ti,ab,kw OR 'food craving':ti,ab,kw OR 'food cue':ti,ab,kw OR 'food cues':ti,ab,kw OR 'food stimuli':ti,ab,kw OR 'high-calorie food':ti,ab,kw OR 'palatable food':ti,ab,kw) AND ('event related potential'/exp OR 'event-related potential':ti,ab,kw OR 'event-related potentials':ti,ab,kw OR ERP:ti,ab,kw OR EEG:ti,ab,kw OR P1:ti,ab,kw OR N1:ti,ab,kw OR N2:ti,ab,kw OR P3:ti,ab,kw OR P300:ti,ab,kw OR LPP:ti,ab,kw OR 'late positive potential':ti,ab,kw OR SPN:ti,ab,kw OR 'stimulus-preceding negativity':ti,ab,kw OR RewP:ti,ab,kw OR 'reward positivity':ti,ab,kw OR ERN:ti,ab,kw OR 'error-related negativity':ti,ab,kw OR Pe:ti,ab,kw OR 'error positivity':ti,ab,kw)** | 6,200 results |
| #3  Depression, Reward Processing, Conflict Monitoring, and ERP | **('depression'/exp OR 'depressive disorder'/exp OR depression:ti,ab,kw OR 'depressive disorder':ti,ab,kw OR 'major depressive disorder':ti,ab,kw OR 'depressive symptoms':ti,ab,kw OR anhedonia:ti,ab,kw) AND ('reward processing':ti,ab,kw OR 'reward responsiveness':ti,ab,kw OR 'reward sensitivity':ti,ab,kw OR 'reward anticipation':ti,ab,kw OR 'reward feedback':ti,ab,kw OR 'conflict monitoring':ti,ab,kw OR 'error monitoring':ti,ab,kw OR 'inhibitory control':ti,ab,kw OR 'cognitive control':ti,ab,kw) AND ('event related potential'/exp OR 'event-related potential':ti,ab,kw OR 'event-related potentials':ti,ab,kw OR ERP:ti,ab,kw OR EEG:ti,ab,kw OR N2:ti,ab,kw OR P3:ti,ab,kw OR P300:ti,ab,kw OR LPP:ti,ab,kw OR 'late positive potential':ti,ab,kw OR SPN:ti,ab,kw OR 'stimulus-preceding negativity':ti,ab,kw OR RewP:ti,ab,kw OR 'reward positivity':ti,ab,kw OR ERN:ti,ab,kw OR 'error-related negativity':ti,ab,kw OR Pe:ti,ab,kw OR 'error positivity':ti,ab,kw)** | 696 results |
| #4  Exercise, Depression/Eating Behavior, and ERP | **('exercise'/exp OR 'motor activity'/exp OR exercise:ti,ab,kw OR 'physical activity':ti,ab,kw OR 'aerobic exercise':ti,ab,kw OR 'resistance exercise':ti,ab,kw OR 'high-intensity interval training':ti,ab,kw OR HIIT:ti,ab,kw OR 'acute exercise':ti,ab,kw OR 'exercise intervention':ti,ab,kw OR 'exercise training':ti,ab,kw) AND ('depression'/exp OR 'depressive disorder'/exp OR depression:ti,ab,kw OR 'depressive symptoms':ti,ab,kw OR 'major depressive disorder':ti,ab,kw OR 'emotional eating':ti,ab,kw OR 'food cue':ti,ab,kw OR 'food cues':ti,ab,kw OR 'food craving':ti,ab,kw OR appetite:ti,ab,kw OR 'eating behavior':ti,ab,kw) AND ('event related potential'/exp OR 'event-related potential':ti,ab,kw OR 'event-related potentials':ti,ab,kw OR ERP:ti,ab,kw OR EEG:ti,ab,kw OR N2:ti,ab,kw OR P3:ti,ab,kw OR P300:ti,ab,kw OR LPP:ti,ab,kw OR 'late positive potential':ti,ab,kw OR SPN:ti,ab,kw OR 'stimulus-preceding negativity':ti,ab,kw OR RewP:ti,ab,kw OR 'reward positivity':ti,ab,kw OR ERN:ti,ab,kw OR 'error-related negativity':ti,ab,kw OR Pe:ti,ab,kw OR 'error positivity':ti,ab,kw)** | 1,921 results |
| #5  Direct-evidence search combining depression, emotional eating/food cues, ERP/EEG, and exercise | **('depression'/exp OR 'depressive disorder'/exp OR depression:ti,ab,kw OR 'depressive disorder':ti,ab,kw OR 'major depressive disorder':ti,ab,kw OR 'depressive symptoms':ti,ab,kw OR anhedonia:ti,ab,kw) AND ('emotional eating':ti,ab,kw OR 'emotional overeating':ti,ab,kw OR 'emotional over-eating':ti,ab,kw OR 'emotional undereating':ti,ab,kw OR 'emotional under-eating':ti,ab,kw OR overeating:ti,ab,kw OR undereating:ti,ab,kw OR 'appetite change':ti,ab,kw OR 'food cue':ti,ab,kw OR 'food cues':ti,ab,kw OR 'food craving':ti,ab,kw OR 'eating behavior':ti,ab,kw) AND ('event related potential'/exp OR 'event-related potential':ti,ab,kw OR 'event-related potentials':ti,ab,kw OR ERP:ti,ab,kw OR EEG:ti,ab,kw OR P1:ti,ab,kw OR N1:ti,ab,kw OR N2:ti,ab,kw OR P3:ti,ab,kw OR P300:ti,ab,kw OR LPP:ti,ab,kw OR 'late positive potential':ti,ab,kw OR SPN:ti,ab,kw OR 'stimulus-preceding negativity':ti,ab,kw OR RewP:ti,ab,kw OR 'reward positivity':ti,ab,kw OR ERN:ti,ab,kw OR 'error-related negativity':ti,ab,kw OR Pe:ti,ab,kw OR 'error positivity':ti,ab,kw) AND ('exercise'/exp OR 'motor activity'/exp OR exercise:ti,ab,kw OR 'physical activity':ti,ab,kw OR 'aerobic exercise':ti,ab,kw OR 'resistance exercise':ti,ab,kw OR 'high-intensity interval training':ti,ab,kw OR HIIT:ti,ab,kw OR 'exercise intervention':ti,ab,kw OR 'exercise training':ti,ab,kw)** | 8 results |

| **Search Query** | **Search strategy** | **amount** |
| --- | --- | --- |
| **Cochrane** | | |
| #1  Depression and Emotional Eating Phenotype | **([mh Depression] OR [mh "Depressive Disorder"] OR depression:ti,ab,kw OR "depressive disorder":ti,ab,kw OR "major depressive disorder":ti,ab,kw OR "depressive symptoms":ti,ab,kw OR anhedonia:ti,ab,kw) AND ("emotional eating":ti,ab,kw OR "emotional overeating":ti,ab,kw OR "emotional over-eating":ti,ab,kw OR "emotional undereating":ti,ab,kw OR "emotional under-eating":ti,ab,kw OR overeating:ti,ab,kw OR "over-eating":ti,ab,kw OR undereating:ti,ab,kw OR "under-eating":ti,ab,kw OR "appetite change":ti,ab,kw OR "increased appetite":ti,ab,kw OR "decreased appetite":ti,ab,kw OR "appetite loss":ti,ab,kw OR "eating behavior":ti,ab,kw)** | 886 results |
| #2  Food Cues, Emotional Eating, and ERP | **("emotional eating":ti,ab,kw OR "binge eating":ti,ab,kw OR "binge eating disorder":ti,ab,kw OR obesity:ti,ab,kw OR overweight:ti,ab,kw OR "food craving":ti,ab,kw OR "food cue":ti,ab,kw OR "food cues":ti,ab,kw OR "food stimuli":ti,ab,kw OR "high-calorie food":ti,ab,kw OR "palatable food":ti,ab,kw) AND ("event-related potential":ti,ab,kw OR "event-related potentials":ti,ab,kw OR ERP:ti,ab,kw OR EEG:ti,ab,kw OR P1:ti,ab,kw OR N1:ti,ab,kw OR N2:ti,ab,kw OR P3:ti,ab,kw OR P300:ti,ab,kw OR LPP:ti,ab,kw OR "late positive potential":ti,ab,kw OR SPN:ti,ab,kw OR "stimulus-preceding negativity":ti,ab,kw OR RewP:ti,ab,kw OR "reward positivity":ti,ab,kw OR ERN:ti,ab,kw OR "error-related negativity":ti,ab,kw OR Pe:ti,ab,kw OR "error positivity":ti,ab,kw)** | 514 results |
| #3  Depression, Reward Processing, Conflict Monitoring, and ERP | **([mh Depression] OR [mh "Depressive Disorder"] OR depression:ti,ab,kw OR "depressive disorder":ti,ab,kw OR "major depressive disorder":ti,ab,kw OR "depressive symptoms":ti,ab,kw OR anhedonia:ti,ab,kw) AND ("reward processing":ti,ab,kw OR "reward responsiveness":ti,ab,kw OR "reward sensitivity":ti,ab,kw OR "reward anticipation":ti,ab,kw OR "reward feedback":ti,ab,kw OR "conflict monitoring":ti,ab,kw OR "error monitoring":ti,ab,kw OR "inhibitory control":ti,ab,kw OR "cognitive control":ti,ab,kw) AND ("event-related potential":ti,ab,kw OR "event-related potentials":ti,ab,kw OR ERP:ti,ab,kw OR EEG:ti,ab,kw OR N2:ti,ab,kw OR P3:ti,ab,kw OR P300:ti,ab,kw OR LPP:ti,ab,kw OR "late positive potential":ti,ab,kw OR SPN:ti,ab,kw OR "stimulus-preceding negativity":ti,ab,kw OR RewP:ti,ab,kw OR "reward positivity":ti,ab,kw OR ERN:ti,ab,kw OR "error-related negativity":ti,ab,kw OR Pe:ti,ab,kw OR "error positivity":ti,ab,kw)** | 80 results |
| #4  Exercise, Depression/Eating Behavior, and ERP | **([mh Exercise] OR [mh "Motor Activity"] OR exercise:ti,ab,kw OR "physical activity":ti,ab,kw OR "aerobic exercise":ti,ab,kw OR "resistance exercise":ti,ab,kw OR "high-intensity interval training":ti,ab,kw OR HIIT:ti,ab,kw OR "acute exercise":ti,ab,kw OR "exercise intervention":ti,ab,kw OR "exercise training":ti,ab,kw) AND ([mh Depression] OR [mh "Depressive Disorder"] OR depression:ti,ab,kw OR "depressive symptoms":ti,ab,kw OR "major depressive disorder":ti,ab,kw OR "emotional eating":ti,ab,kw OR "food cue":ti,ab,kw OR "food cues":ti,ab,kw OR "food craving":ti,ab,kw OR appetite:ti,ab,kw OR "eating behavior":ti,ab,kw) AND ("event-related potential":ti,ab,kw OR "event-related potentials":ti,ab,kw OR ERP:ti,ab,kw OR EEG:ti,ab,kw OR N2:ti,ab,kw OR P3:ti,ab,kw OR P300:ti,ab,kw OR LPP:ti,ab,kw OR "late positive potential":ti,ab,kw OR SPN:ti,ab,kw OR "stimulus-preceding negativity":ti,ab,kw OR RewP:ti,ab,kw OR "reward positivity":ti,ab,kw OR ERN:ti,ab,kw OR "error-related negativity":ti,ab,kw OR Pe:ti,ab,kw OR "error positivity":ti,ab,kw)** | 259 results |
| #5  Direct-evidence search combining depression, emotional eating/food cues, ERP/EEG, and exercise | **([mh Depression] OR [mh "Depressive Disorder"] OR depression:ti,ab,kw OR "depressive disorder":ti,ab,kw OR "major depressive disorder":ti,ab,kw OR "depressive symptoms":ti,ab,kw OR anhedonia:ti,ab,kw) AND ("emotional eating":ti,ab,kw OR "emotional overeating":ti,ab,kw OR "emotional over-eating":ti,ab,kw OR "emotional undereating":ti,ab,kw OR "emotional under-eating":ti,ab,kw OR overeating:ti,ab,kw OR undereating:ti,ab,kw OR "appetite change":ti,ab,kw OR "food cue":ti,ab,kw OR "food cues":ti,ab,kw OR "food craving":ti,ab,kw OR "eating behavior":ti,ab,kw) AND ("event-related potential":ti,ab,kw OR "event-related potentials":ti,ab,kw OR ERP:ti,ab,kw OR EEG:ti,ab,kw OR P1:ti,ab,kw OR N1:ti,ab,kw OR N2:ti,ab,kw OR P3:ti,ab,kw OR P300:ti,ab,kw OR LPP:ti,ab,kw OR "late positive potential":ti,ab,kw OR SPN:ti,ab,kw OR "stimulus-preceding negativity":ti,ab,kw OR RewP:ti,ab,kw OR "reward positivity":ti,ab,kw OR ERN:ti,ab,kw OR "error-related negativity":ti,ab,kw OR Pe:ti,ab,kw OR "error positivity":ti,ab,kw) AND ([mh Exercise] OR [mh "Motor Activity"] OR exercise:ti,ab,kw OR "physical activity":ti,ab,kw OR "aerobic exercise":ti,ab,kw OR "resistance exercise":ti,ab,kw OR "high-intensity interval training":ti,ab,kw OR HIIT:ti,ab,kw OR "exercise intervention":ti,ab,kw OR "exercise training":ti,ab,kw)** | 3 results |
